# Clinical Relevance and Management of Recurrent Laryngeal Nerve Inlet Zone Lymph Nodes Metastasis in Papillary Thyroid Cancer

**DOI:** 10.3389/fendo.2021.653184

**Published:** 2021-07-22

**Authors:** Guibin Zheng, Guochang Wu, Haiqing Sun, Chi Ma, Yawen Guo, Dongmin Wei, Wenbin Yu, Haitao Zheng, Xicheng Song

**Affiliations:** ^1^ Department of Thyroid Surgery, The Affiliated Yantai Yuhuangding Hospital of Qingdao University, Yantai, China; ^2^ Department of Otorhinolaryngology, Qilu Hospital of Shandong University, NHC Key Laboratory of Otorhinolaryngology (Shandong University), Jinan, China; ^3^ Key Laboratory of Carcinogenesis and Translational Research (Ministry of Education), Department of Head and Neck Surgery, Peking University Cancer Hospital & Institute, Beijing, China; ^4^ Department of Otolaryngology-Head and Neck Surgery, The Affiliated Yantai Yuhuangding Hospital of Qingdao University, Yantai, China

**Keywords:** papillary thyroid cancer, recurrent laryngeal nerve inlet zone, lymph nodes metastasis, recurrent nodal disease, neck dissection

## Abstract

**Background:**

Recurrent nodal disease often occurs in recurrent laryngeal nerve inlet zone (RLNIZ), leading to difficult surgical management.

**Methods:**

Medical records of 947 patients with PTC and 33 patients with recurrent PTC were retrospectively reviewed. Totally 169 sides of RLNIZ dissection in 152 patients (17 cases were bilateral and 135 cases were unilateral) with primary surgery and 4 patients with structural recurrent disease were included for the analysis.

**Results:**

The rate of lymph node metastasis in RLNIZ was 31.3% (47/150). The incidence of transient hypoparathyroidism was 5.9% and no RLN injury and permanent hypoparathyroidism occurred. RLNIZ lymph nodes metastasis (LNM) was significantly associated with age <45 years, larger tumor size, number of CNLNM, and lateral node metastasis. CNLNM and lateral node metastasis were independent risk factors for RLNIZ LNM. Recurrent nodal disease in RLNIZ was identified in four of 33 patients and permanent recurrent laryngeal nerve (RLN) injury was observed in one of four patients.

**Conclusion:**

Lymph nodes in RLNIZ are usually involved in patients with heavy tumor burden and can be removed safely at initial surgery. Once central or lateral LNM was confirmed preoperatively or intraoperatively, RLNIZ lymph node dissection should be carefully performed to reduce the rate of structural recurrence in the central compartment.

## Introduction

Papillary thyroid cancer (PTC) is an indolent malignant tumor with low mortality. Central lymph node metastasis has been reported in approximately 31.5–50% of patients with PTC at initial surgery (1–4). In spite of low mortality, 3–7.9% of patients have loco-regional recurrence within 5 years after initial surgery (5–9). Of these patients, structural recurrence in the central compartment accounts for 20–55.6% of all loco-regional recurrences (4, 7, 9–11). This proportion of early nodal recurrence reflects the inadequacy of initial surgical management (12, 13). Surgical reintervention for central compartment recurrences especially for RLN inlet region is more challenging and may substantially increase the risk of recurrent laryngeal nerve (RLN) injury as well as permanent hypoparathyroidism. The latter results from tissue scarring, and disruption of normal anatomy may lead to an overall reduced quality of life for the survivor (11).

Clayman et al. (14) reported precise locations of recurrent lymph nodes within the central compartment and that 22.4% (47/210) of patients with recurrent disease had metastatic nodal disease in the RLN inlet. Such patients had high risk for treatment. However, this study did not have a clear definition of region for metastatic nodal disease in RLN inlet. The RLN inlet is closely surrounded by the thyroid gland, esophagus, trachea and cricoid cartilage, which form a latent space adjacent to RLN inlet, named RLN inlet zone (RLNIZ). RLNIZ belongs to paratracheal subregion of central compartment and lacks adipose tissue. Lymph nodes in RLNIZ are small and uncommon. Although lymph nodes in RLNIZ are part of paratracheal lymph nodes, these lymph nodes may be easily omitted during surgery or may be intentionally overlooked for concerns of RLN and parathyroid gland injury. Detailed investigation of lymph node metastasis in RLNIZ would provide crucial information for optimizing the initial surgical intervention. However, there is limited evidence focused on metastatic nodal disease in RLNIZ in patients with PTC.

This study aims to explore the prevalence and characteristics of metastatic lymph nodes in RLNIZ in patients with PTC at primary surgery and to determine the surgical management of patients with structural recurrence at RLNIZ.

## Methods And Study Patients

### Patients

We retrospectively reviewed the medical records of 947 patients with PTC and 33 patients with recurrent PTC between July 1, 2017 and June 30, 2018 in the Department of Thyroid Surgery and Department of Otorhinolaryngology-Head & Neck Surgery at the Affiliated Yantai Yuhuangding Hospital of Qingdao University. Recurrence was determined by fine-needle aspiration biopsy in all 33 patients with previous history of surgery for PTC. Structural recurrent disease in RLNIZ was evaluated by CT scan preoperatively and confirmed by histopathological examination postoperatively. Ipsilateral central neck dissection (CND) was performed routinely in all patients with PTC at primary surgery. Patients with pathological examination of tissues in RLNIZ removed separately were included in the study. This cohort is comprised of 152 patients who underwent primary surgery for PTC and four patients with structural recurrent disease in RLNIZ. The study protocol was approved by the Committee of Ethics in Research of our institution.

### Anatomy of RLNIZ

**Figure 1:**
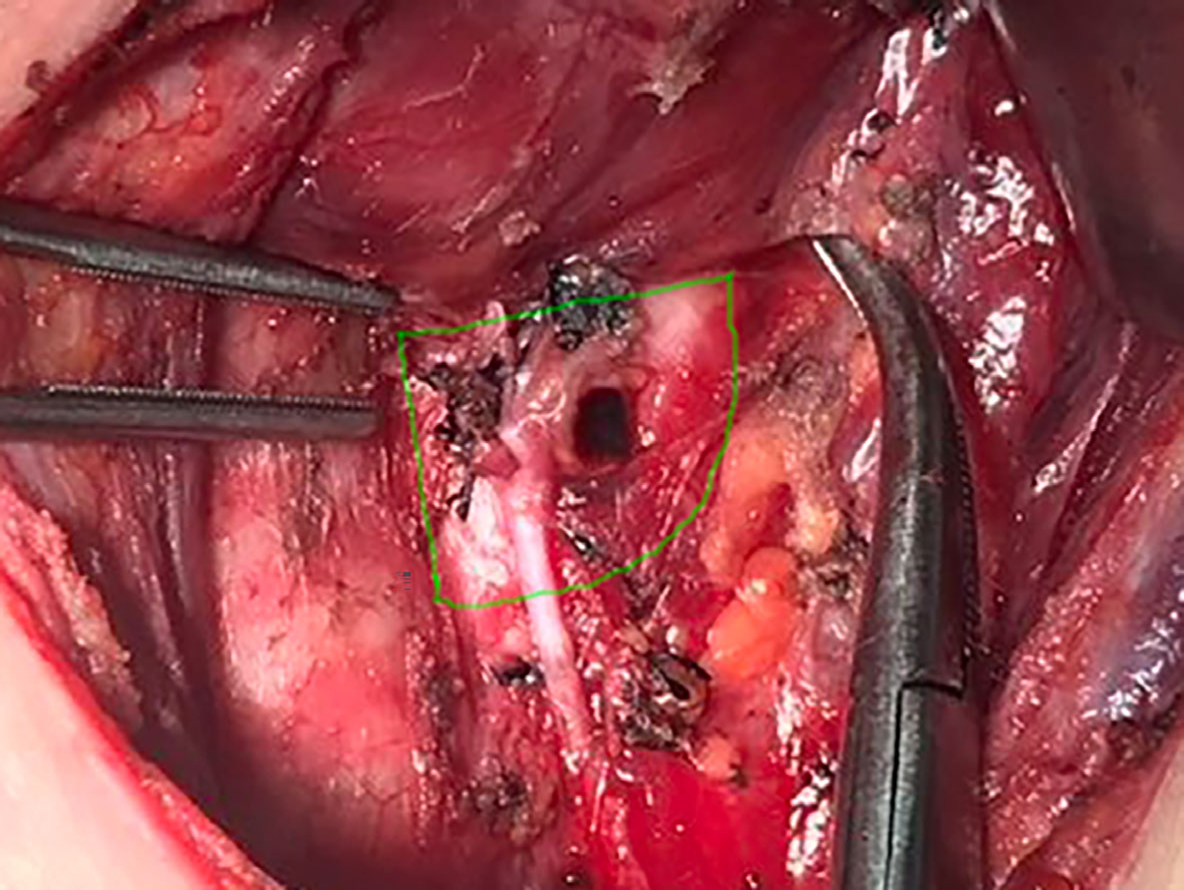
The region of RLNIZ. Green line showed the region of RLNIZ needed to be dissected during surgery.

RLNIZ refers to the convergence area of important anatomical structures related to the thyroid surgery including RLN, Berry ligament, branches of inferior thyroid artery that enter the thyroid lobe near the RLN inlet and superior parathyroid gland (SPG). In this region, Berry ligament firmly anchors the thyroid gland to the trachea and then forms a latent space lacking adipose tissue. The boundaries of RLNIZ are: cricoid cartilage, located superiorly; the upper part of trachea, located medially; and the lateral side of thyroid gland, located laterally. There is no clear inferior boundary of RLNIZ due to the variability of the horizontal segment of inferior thyroid artery traversing RLN. Therefore, based on the anatomical structures and the difficulty of surgical intervention, we defined RLNIZ as a 1-cm radius space around RLN inlet for the purpose of this study (**Figure 1**).

### Surgery Strategies and Removal of RLNIZ Lymph Nodes

The surgery strategies for PTC were selected according to the guidelines in China as follows: Unilateral thyroidectomy plus isthmectomy was performed for unilateral PTC. Total thyroidectomy was performed for bilateral PTC or unilateral PTC with tumor size over 4 cm. Prophylactic central neck dissection (CND) was routinely performed on the tumor side. Lateral neck dissection was performed when metastatic lymph nodes on the lateral neck were confirmed preoperatively by fine needle aspiration biopsy and wash-out thyroglobulin (Tg) or by intraoperative frozen biopsy.

CND was performed after thyroidectomy. Removal of RLNIZ lymph nodes was performed under a direct vision of RLN as the final step of CND. RLNIZ was meticulously dissected and explored during primary surgery. Any tissue identified was deliberately removed, labeled as the RLNIZ lymph nodes and sent for paraffin pathology examination separately. In patients with recurrence disease, RLNIZ dissection was only performed with the confirmation of structural recurrence in RLNIZ by preoperative ultrasonography and/or enhanced CT scan in order to decrease the risk of RLN injury. All the surgeries were performed by high-volume surgeons.

### Data Collection and Statistical Analysis

As RLNIZ dissection was performed bilaterally in some patients with bilateral PTC, the clinicopathological data were collected for analysis based on the sides of RLNIZ removal rather than the number of patients. SPSS v. 20.0 (SPSS, Inc., Chicago, IL, USA) was used for all statistical analyses. Categorical variables were tested with Chi-square test or Kruskal–Wallis test. Continuous variables were analyzed using the Student’s t-test or Mann–Whitney U test. Characteristics found to be significantly different between groups in the univariate analysis were included in the multivariate logistic regression analysis. Differences were considered significant when *P <*0.05.

## Results

### Characteristics of Patients Who Underwent Primary Surgery

Unilateral RLNIZ dissection was performed in 135 patients, and bilateral RLNIZ dissection was performed in 17 patients with bilateral PTC. Thus, totally 169 sides of RLNIZ dissection were performed in 152 patients. Of these patients, 83 (54.6%) were found to have central neck lymph node metastasis (CNLNM). Of 169 sides that had RLNIZ removed, thirteen (7.7%) were merely fibrous adipose tissue, nine (5.3%) had thyroid tissue, and three (1.8%) were confirmed as parathyroid glands by histopathology. Lymph nodes in RLNIZ were harvested in 150 sides of 136 patients (**Table 1**). Metastatic disease to RLNIZ was observed in 47 out of 150 sides (31.3%). The median number and size of lymph nodes in RINIZ was 1 (range: 1–4) and 0.25 cm (range: 0.1–0.6 cm), respectively. Of the 150 sides of RLNIZ, eight (5.3%) had only RLNIZ lymph node metastasis in the central compartment.

**Table 1:** Clinical characteristics of patients with primary surgery for PTC who had lymph nodes in RLNIZ.

Characteristics	RLNIZ+ (%) [n = 47]^§^	RLNIZ− (%) [n = 103] ^§^	*P-value*
Sex			0.325^*^
Female	31 (66.0)	76(73.8)	
Male	16 (34.0)	27(26.2)	
Age			<0.001^*^
<45 years	31 (66.0)	31 (30.1)	
≥45 years	16 (34.0)	7 2(69.9)	
Tumor location			0.994
Upper 1/3	7 (14.9)	15 (14.6)	
Middle 1/3	33 (70.2)	72 (69.9)	
Lower 1/3	7 (14.9)	16 (15.5)	
Thyroiditis			0.611^*^
Present	14 (29.8)	35 (34.0)	
Absent	33 (70.2)	68 (66.9)	
Capsular invasion	32 (68.1)	58 (56.3)	0.172^*^
Tumor size(cm)	1.2 ± 0.8	0.8 ± 0.5	0.005^†^
Multifocality			0.628^*^
Yes	8 (17.0)	21 (25.6)	
No	39 (83.0)	82 (79.6)	
BRAF^V600E^ gene			0.769^¶^
Mutant	24 (51.1)	59 (57.3)	
Wild type	13 (27.7)	24 (23.3)	
Undetected	10 (21.3)	20 (19.4)	
No. of central nodes removed	11.5 ± 5.2	10.8 ± 4.5	0.355^†^
No. of CNLNM	6.1 ± 4.7	1.1 ± 2.1	<0.001^†^
Lateral nodes metastasis			<0.001^*^
Present	18 (38.3)	3 (2.9)	
Absent	29 (61.7)	100 (97.1)	
No. of lateral nodes removed	28.8 ± 9.6	23.7 ± 12.9	0.417^†^
No. of LNLNM	6.1 ± 4.2	1.6 ± 1.2	0.017^‡^

^*^Chi-square test. ^†^Student’s t-test. ^‡^Mann–Whitney U test. ^¶^Kruskal–Wallis test. ^§^Number of the RLNIZ sides. + positive for metastatic disease; − negative for metastatic disease. RLNIZ, recurrent laryngeal nerve inlet zone; CNLNM, central neck lymph nodes metastasis; NLNM, lateral neck lymph node metastasis.

Univariate analysis was performed between the group with (n = 47) and without (n = 103) RLNIZ lymph node metastasis. Metastatic disease to RINIZ was associated with age<45 years (66.0 *vs*. 30.1%, *P* < 0.001, larger tumor size (1.2 ± 0.8 *vs*. 0.8 ± 0.5, *P* = 0.005), number of CNLNM (6.1 ± 4.7 *vs*. 1.1 ± 2.1, *P* < 0.001) and lateral node metastasis (38.3 *vs*. 2.9%, *P* < 0.001) as shown in **Table 1**. There were no significant effects of sex, tumor location, thyroiditis, capsular invasion, and BRAF^V600E^ mutation. Multivariate analyses showed a statistically significant difference in age<45 years (*P* = 0.014), number of CNLNM (*P* < 0.001), and lateral node metastasis (*P* = 0.019) as shown in **Table 2**.

**Table 2:** Multivariable analysis of RLNIZ lymph node metastasis in patients with primary surgery for PTC.

Characteristics	OR	95% CI	*P*
Age<45 years	0.274	0.097–0.771	0.014
Tumor size	1.343	0.613–2.944	0.462
No. of CNLNM	1.674	1.353–2.072	<0.001
Lateral nodes metastasis	7.122	1.383–36.668	0.019

RLNIZ, recurrent laryngeal nerve inlet zone. OR, odds ratio; CI, confidence interval. CNLNM, central neck lymph nodes metastasis.

Of these 152 patients, no RLN injury or permanent hypoparathyroidism was observed. Temporary hypoparathyroidism occurred in nine out of 152 patients (5.9%). Nine patients with total thyroidectomy and lateral neck dissection were treated with adjuvant radioactive iodine (RAI) postoperatively. The median follow-up time was 16 months (range, 12–28), no recurrent disease was found by US or serum Tg.

### Characteristics of Patients With Recurrent Disease in RLNIZ

**Figure 2:**
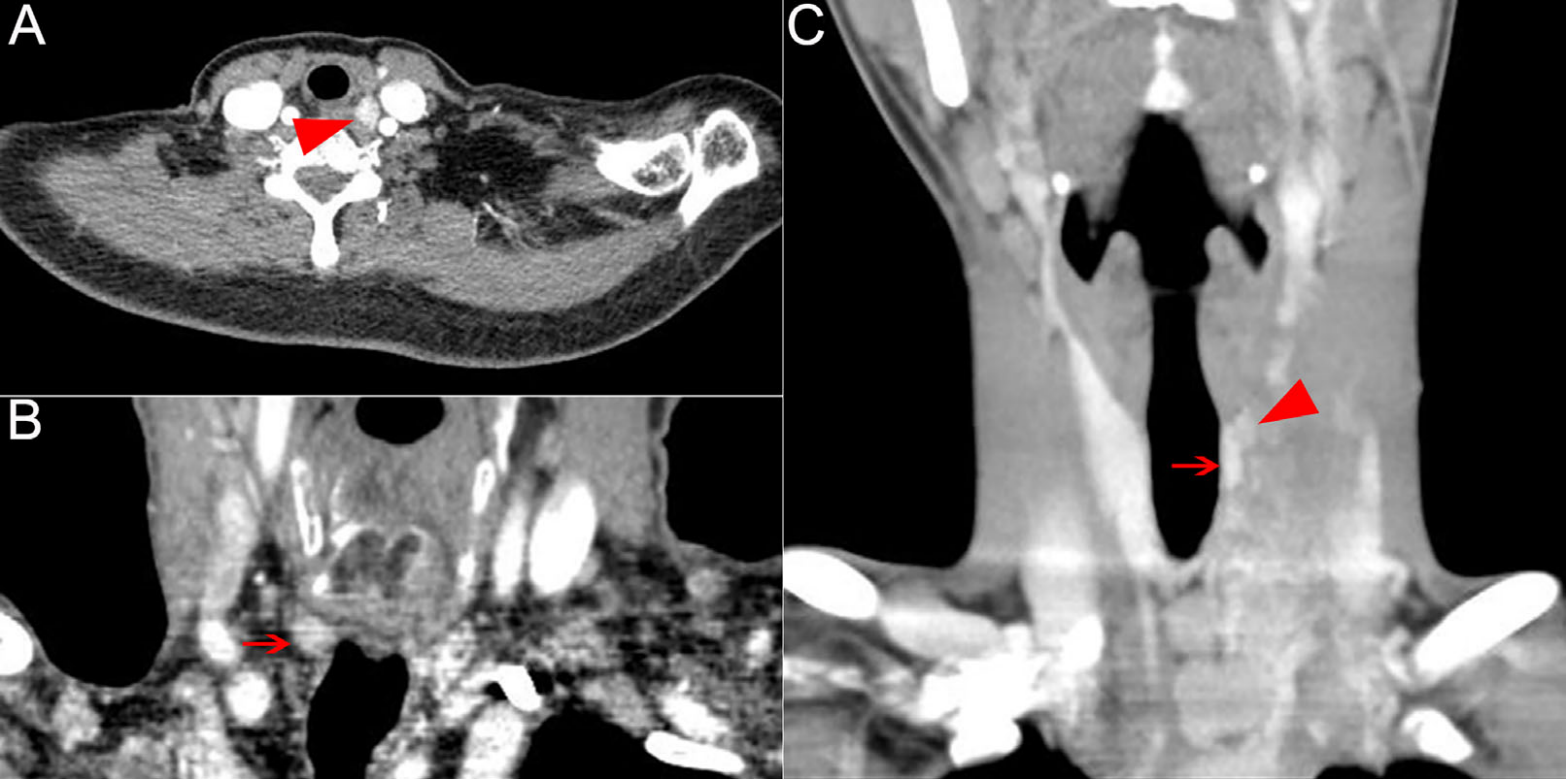
Recurrent nodal disease in RLNIZ diagnosed by enhanced computer tomography. **(A)** Recurrent nodal disease in RLNIZ in case 1. **(B)** Residual thyroid tissue in RLNIZ without tumor foci. **(C)** Recurrent nodal disease and residual thyroid tissue with tumor foci in RLNIZ in case 4. Red arrowhead: recurrent nodal disease; red arrow: residual thyroid tissue. RLNIZ, recurrent laryngeal nerve inlet zone.

During this study period, recurrent disease in RLNIZ was found in four of 33 patients (12.1%) as shown in **Table 3** and **Figure 2**. The information of the primary tumors of those four patients was unknown because their initial surgery for PTC was performed in other institutions. Recurrent disease in RLNIZ was examined by enhanced CT scan and confirmed by histopathological examination after surgery. In case 1 (**Figure 2A**), recurrent disease in RLNIZ was the major finding by preoperative ultrasonography. Permanent RLN injury occurred in one of four patients (25%). No temporary RLN paralysis or hypocalcemia was noted. RAI was given to those four patients; none of them showed any evidence of recurrent disease during the follow-up period.

**Table 3:** Characteristics of patients with recurrent nodal disease in RLNIZ.

Case number	Age/sex	Time to relapse	Surgery	Location and size of recurrent nodal disease in RLNIZ	Complications
1	59/F	6 years	Left CND; Left LND	Left 1.9 × 1.0 cm	No
2	59/M	2 years	Bilateral CND; Right LND;	Left 0.9 × 0.7cm	No
3	46/F	10 years	Bilateral CND; Bilateral LND; Parapharyngeal lymph nodes dissection	Right 0.5 × 0.5 cm	Right vocal cord paralysis
4	16/M	6 months	Residual thyroidectomy; Bilateral CND; Bilateral LND	Left 0.7 × 0.5cm	No

RLNIZ, recurrent laryngeal nerve inlet zone; CND, central neck dissection; LND, lateral neck dissection; F, female; M, male.

## Discussion

RLNIZ, a convergence zone of several important anatomical structures, is a region often involved with recurrent nodal disease of PTC that is difficult to manage because of the dense adhesion (14, 15). To the best of our knowledge, RLNIZ is defined based on a surgical experience, and the standardized definition remains lacking. However, surgeons should pay more attention to this region of primary surgery to reduce the risk of RLN injury, as this region may have surgeons perform subtotal thyroidectomy or unintentionally neglect small lymph nodes. In this study, we referred to RLNIZ as a 1-cm radius space around RLN inlet for several reasons. First, there is no clear inferior boundary of RLNIZ due to the variability of the horizontal segment of inferior thyroid artery traversing RLN; second, there are constant small vessels that enter the gland within 1 cm region near the RLN inlet, and bleeding usually occurs in this region when dissecting RLN and excising lymph nodes; and third, small gland and lymph nodes are usually left in the 1-cm radius space due to concern for RLN injury. Given that recurrent nodal disease in RLNIZ is not rare (14), more attention should be paid to the dissection of lymph nodes in RLNIZ for primary surgery.

**Figure 3:**
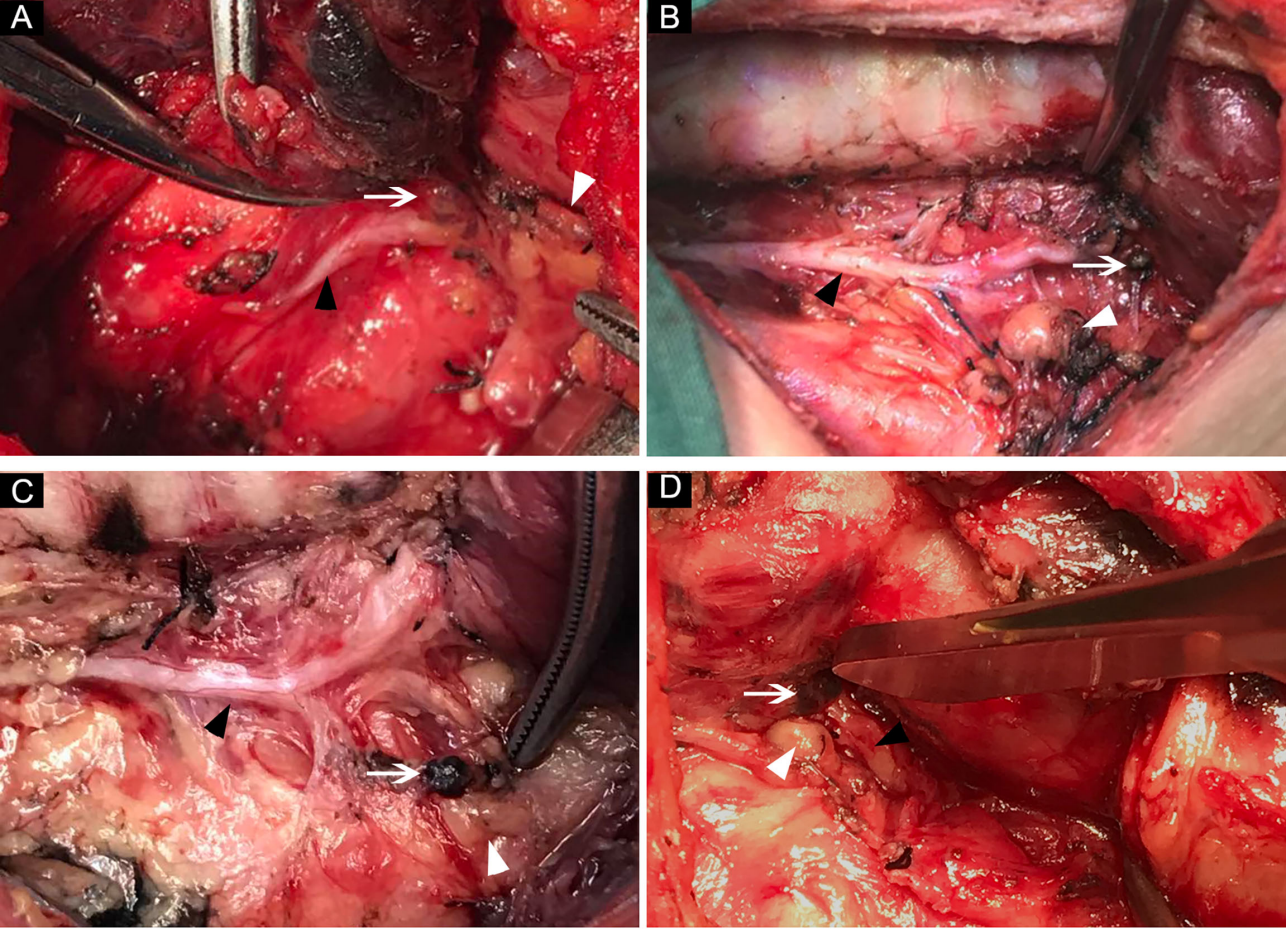
The common locations of lymph nodes in RLNIZ. **(A)** Lymph node located medially to the RLN inlet; **(B)** small lymph node located laterally to the RLN inlet; **(C)** lymph node located far from RLN inlet due the traction; **(D)** lymph node and SPG located closely to each other at RLNIZ. White arrow: lymph nodes in RLNIZ; black arrow head: RLN; white arrowhead: superior parathyroid gland. RLNIZ, recurrent laryngeal nerve inlet zone; RLN, recurrent laryngeal nerve; SPG, superior parathyroid gland.

Our results found that lymph nodes in RLNIZ were detected in 89.5% (136/152) of patients with PTC at initial surgery. Of the 150 RLNIZ sides removed, the incidence of RLNIZ LNM was 31.3%, which was not rare. The metastatic rate of lymph nodes in RLNIZ was lower than that (46.84%) reported by Tian et al. (15), which might be due to the different definitions of RLNIZ (0.5 cm from the outer edge of the lymph node to the RLN entrance point by Tian et al.) and sample sizes between the studies. The common locations of lymph nodes in RLNIZ were shown in **Figure 3**. As for the patients with recurrent disease in the central compartment, the rate of recurrent disease to RLNIZ was 12.1%.

More importantly, RLNIZ LNM was correlated with several poor prognostic factors, including larger tumor size, increased number of CNLNM, and lateral node metastasis. Thus, it means that lymph nodes in RLNIZ were usually involved in patients with heavy tumor burden. Given that metastatic lymph nodes in RLNIZ were usually too small to be detected preoperatively at primary surgery, the abilities of those related risk factors predicting RLNIZ LNM were studied. As shown in **Table 4**, patients with CNLNM and lateral node metastasis were respectively 4.8 and 3.8 times more likely to suffer from RLNIZ LNM. In addition, RLNIZ LNM was also associated with age less than 45 years. This result agreed with that of a study by Wang et al. (16) which showed an increased lymph node positivity among young patients with PTC. Although older age was considered as a high risk factor for decreased cancer-specific survival, patients younger than 45 years had worse outcomes than older patients within stage II (AJCC staging protocol) (17). Those results indicated that RLNIZ LNM would more likely occur in patients with PTC who suffer heavy disease burden, especially in patients younger than 45 years. Therefore, more attention should be paid to the dissection of lymph nodes in RLNIZ to reduce the risk of structural recurrence in the central compartment in those patients.

**Table 4:** Ability of age<45 years, tumor size>1 cm, potential CNLNM and lateral nodes metastasis to predict RLNIZ lymph nodes metastasis.

	Sensitivity	Specificity	PPV	NPV	LR+	LR-
Age<45 years	50	81.8	66	70	2.7	0.6
Tumor size >1 cm	44.1	76.9	55.3	68	1.9	0.7
Further CNLNM^*^	51.3	89.2	83	64.1	4.8	0.8
Lateral nodes metastasis	85.7	77.5	38.3	97.1	3.8	0.2

RLNIZ, recurrent laryngeal nerve inlet zone; CNLNM, central neck lymph nodes metastasis.

PPV, positive predictive value; NPV, negative predictive value.

LR+, positive likelihood ratio; LR−, negative likelihood ratio.

^*^Patients with only metastatic disease to RLNIZ in central compartment were excluded.

It must be noted that RLNIZ dissection may be deliberately omitted by un-experienced surgeons in attempt to reduce the morbidity of RLN and parathyroid gland injury. A study by Zhang et al. (18) showed that the major mechanism of RLN injury was thermal injury, at a distal 1-cm below the inlet of RLN, during thyroidectomy. Liu et al. (19) reported that excessive stretch of thyroid lobe played an important role in RLN injury near the entry point of RLN, whereas, RLN injury in this region usually occurred in the procedure of thyroidectomy rather than lymph nodes dissection. In our cohort, no RLN injury during primary surgery was observed. Hypoparathyroidism should alarm the surgeon in the procedure of RLNIZ dissection. About 80% of SPGs were generally located within a 2-cm region about 1 cm above the crossing point of RLN and inferior thyroid artery, which could be easily identified and then protected (20). Rodrigues et al. (21) reported that 11.1% right SPG were located medially to RLN. Hence, some of SPGs can be found in RLNIZ (**Figure 3D**), which are more difficult to protect. In our cohort, 1.8% of SPGs were inadvertently removed during RLNIZ dissection, which may add to the risk of permanent hypothyroidism in patients undergoing bilateral CND. Transient hypoparathyroidism occurred in nine of 152 patients (5.9%), and no permanent hypoparathyroidism was noted after the primary surgery. Therefore, RLNIZ dissection can be performed safely by experienced surgeons at initial surgery.

In order to evaluate the significance of RLNIZ node metastasis, we included patients with recurrence disease in the same period time of the study because of the relatively indolent biological behavior of PTC which needed a long period of follow-up. In our cohort, the rate of recurrence of nodal disease in RLNIZ was 12.1%, which was not rare. Vocal cord paralysis was a major concern in those patients who needed a surgical reintervention in the central compartment. About 2–17.8% permanent RLN injury occurs in CND revision (11, 14). In our cohort, one of four patients (25%) with structural recurrence in RLNIZ suffered permanent vocal cord paralysis after reintervention for CND. Tian et al. (15) reported two cases of structural recurrence in RLNIZ, and both resulted in hoarseness after reoperation. Management of recurrent disease in RLNIZ requires meticulous dissection of the RLN. Intraoperative RLN monitoring could aid in nerve identification and protection (22). Given the tight adhesion and fibrosis in RLNIZ, only when recurrent nodal disease in RLNIZ is diagnosed preoperatively should RLNIZ dissection be performed in order to decrease the risk for iatrogenic RLN injury. Surgical approach from the lateral side of strap muscles may be optimal to access the RLNIZ.

Although no hypoparathyroidism was observed in the four patients undergoing reintervention in our study, hypoparathyroidism is still a potential complication in revised CND. The incidence of temporary and permanent hypoparathyroidism in reinterventions is reported to be 46.3–60% and 2–4.6%, respectively (11, 23). Since SPG is located near RLNIZ and is vulnerable to injury during removal of recurrent disease in RLNIZ, only recurrent disease or suspected lymph nodes in RLNIZ should be excised to decrease the risk of permanent hypoparathyroidism, on the premise that SPG may not be identified intraoperatively. When SPG is identified or auto-transplanted intraoperatively, any adipose tissues in or near RLNIZ should be excised radically.

In the evaluation of structural recurrence in RLNIZ, it is difficult to distinguish between residual thyroid tissue from metastatic lymph nodes (**Figure 2B**). Usually, thyroid remnants in RLNIZ were left by some surgeons with the purpose of reducing the morbidity of RLN injury at initial surgery. However, the presence of thyroid remnants is a substantial risk factor for predicting central compartment recurrence after salvage CND (14). In patients undergoing reintervention, 88.6% of thyroid remnants contained residual malignancy (14), which may add to the risk of metastatic disease to RLNIZ (**Figure 2C**) and invasion to RLN (11). Thus, given that the incidence of residual malignancy is high in the patients undergoing reintervention, total thyroidectomy of a lobe should be performed with the assurance of surgical safety.

This study has some limitations. Firstly, this is a retrospective study with relatively small sample, which might bias the findings of the current study. Secondly, the follow-up time was relatively short to precisely evaluate the effect of RLNIZ dissection on the recurrence of PTC after initial surgery. Thus, a larger well-designed prospective study with longer follow-up should be performed to further confirm the significance of RLNIZ dissection in PTC. Thirdly, the relationship between RLNIZ LNM and pathological subtype of PTC was not evaluated since such data could not be obtained from the pathology reports of these study patients. Highly invasive subtypes of PTC may contribute to the accuracy of the analysis for the relationships between RLNIZ LNM and clinicopathological factors. Fourthly, as the information of primary tumors of four patients with recurrent disease in RLNIZ was unknown, the correlation between primary tumor and recurrent nodal disease in RLNIZ could not be evaluated. Lastly, the number of patients with recurrent PTC disease admitted to our institution was small which may explain the lower rate of the recurrent disease in RLNIZ compared to results by Clayman et al. (14). This may lead to undervaluation of the morbidity of complications when reinterventions are needed for recurrent disease in RLNIZ.

In summary, this study provided valuable information regarding the involvement of RLNIZ lymph nodes in patients with PTC at initial surgery. Although the lymph nodes in RLNIZ adjacent to important anatomical structures such as RLN and SPG, RLNIZ dissection can be performed safely by experienced surgeons. The study results suggest that RLNIZ LNM is associated with several poor prognostic factors, and CNLNM and lateral node metastases were highly predictive of RLNIZ LNM. This is helpful for surgeons in optimizing the extent of CND at initial surgery in the patients with heavy tumor burden with a goal of reducing recurrence rate. Structural recurrent disease in RLNIZ is of substantial risk for RLN injury which may be avoided by intentional RLNIZ dissection at initial surgery. Studies with long-term follow-up are needed to affirm conclusions on outcomes and prognosis.

## Data Availability Statement

The raw data supporting the conclusions of this article will be made available by the authors, without undue reservation.

## Ethics Statement 

The studies involving human participants were reviewed and approved by the Committee of Ethics in Research of Affiliated Yantai Yuhuangding Hospital of Qingdao University. The patients/participants provided their written informed consent to participate in this study.

## Author Contributions

GZ, XS, WY, and HZ contributed to conception and design of the study. GW organized the database. HS performed the statistical analysis. GZ wrote the first draft of the manuscript. CM, YG, and DW wrote sections of the manuscript. All authors contributed to the article and approved the submitted version.

## Funding

This work was funded by the Taishan Scholars Program of Shandong Province (NO.ts20190991).

## Conflict of Interest

The authors declare that the research was conducted in the absence of any commercial or financial relationships that could be construed as a potential conflict of interest.

## Abbreviations

CND, central neck dissection; CNLNM, central neck lymph nodes metastasis; CNs, carbon nanoparticles; CT, computer tomography; LNM, lymph nodes metastasis; PTC, papillary thyroid cancer; RAI, adjuvant radioactive iodine; RLN, recurrent laryngeal nerve; RLNIZ, recurrent laryngeal nerve inlet zone; SPG, superior parathyroid gland; Tg, thyroglobulin.
